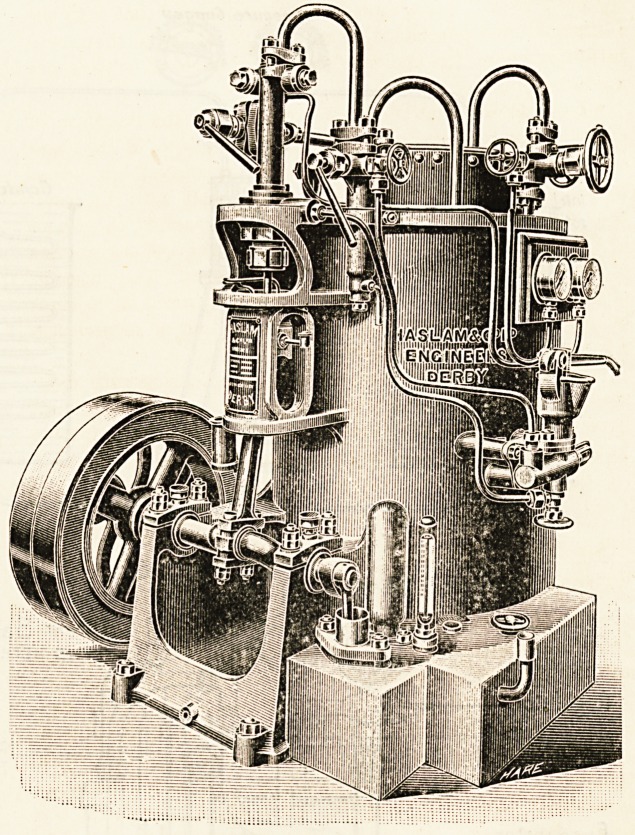# Modern Hospital and Institutional Fittings.—III

**Published:** 1903-12-05

**Authors:** 


### HOSPITAL ADMINISTRATION.

### CONSTRUCTION AND ECONOMICS.

### MODERN HOSPITAL AND INSTITUTIONAL FITTINGS.—III.

REFRIGERATION AND COLD STORAGE.

(*Concluded from page* 105.)

The compression system of mechanical refrigeration is now almost entirely employed, for as we have previously shown, the co-efficient of performance is greater than that of other methods and the apparatus requires less room. Many of the leading hotels and hospitals employ this process, while for breweries, ice factories, meat freezing works, etc., it is used most extensively. The choice of the chemical refrigerating agent has before been touched upon. On account of the noxiousness of ammonia, and the possible danger involved in its use, objections which now are of little or no consequence owing to certain technical improvements, carbonic acid has come to be the agent most extensively used; although ammonia is undoubtedly the most economical and efficient.

Even in tropical countries, where the water used to cool the compressed gas is at a comparatively high temperature, these machines are found to work wonderfully well. Just of late sulphurous acid gas machines have been revived somewhat owing to the fact that their patent has expired, and so they can be installed cheaply, though their relative efficiency is a good deal less than those using the other agents.

There are many first-class firms producing machines of a high order, and we have had the opportunity of witnessing the various installations at work. Messrs. H. J. West & Co., of the Stamford Works, Southwark Bridge Road, build a very efficient machine which is spoken of most highly by those who have it in practical use, and we were well assured of its practical value. Messrs. J. and E. Hall have been for many years the pioneers of refrigerative machinery, and have placed installations in three of our leading hospitals (St. George's, University, and London). Their machines answer well to all the claims that the firm makes for them, and this notwithstanding the fact that in building the plant they had many engineering difficulties to overcome. It is evidently of great importance in the building of any new institution that may require a refrigerative plant, that the architect should early bear this fact in mind, or great difficulties may arise afterwards. This is well illustrated by our noticing that in one of our hospitals hot-water pipes ran through one of the cold rooms! They, of course, are well insulated, yet detract greatly from the efficiency of the refrigeration, and this inconvenience would not have happened had the planning of cold storage been earlier attended to.

The Haslam Foundry and Engineering Company, another firm in the first rank, have supplied a leading hospital (St. Thomas's) with their plant, and we found the cooling of stores (40° Fahr.) and the mortuary (35° Fahr.) splendidly performed, though the brine pipes have to travel a long way to their destination. An ice tank was also viewed at work, where ice was being rapidly made by the cold brine circulating around metal moulds which are constantly kept rocking in order to produce clear ice

A ton can thus be produced at the cost of about 4s. 6d., while ice delivered to the hospital would cost about 25s. per ton.

Messrs. Ransomes and Rapier, of London and Ipswich, are installing one of their "Simplex" ammonia absorption machines in another large hospital (Middlesex). An advantage of this system is that it involves the motion of hardly any machinery except that of the brine pump, and hence little disturbance will be noticed in the wards above.

The majority of our hospitals at present, though, have not seen their way to avail themselves of the advantages of mechanical refrigeration, but as re-building and extension of these institutions take place, we shall doubtless see an advance in this direction. Refrigeration has a vast future before it, and industries will in time be created and enhanced by its means. A visit to the magnificent London Docks Cold Storage, where "one can see coal go in at one end and ice come out at the other," and where thousand upon thousand of meat carcases are stored and kept for human consumption, will strike everyone with the wonderful possibilities lying before this branch of engineering.

In time of siege of what national importance might not these cold stores be? In a few generations, refrigeration will undoubtedly have made such strides that wonders now undreamt of will have been accomplished.

## Figures and Tables

**Figure f1:**
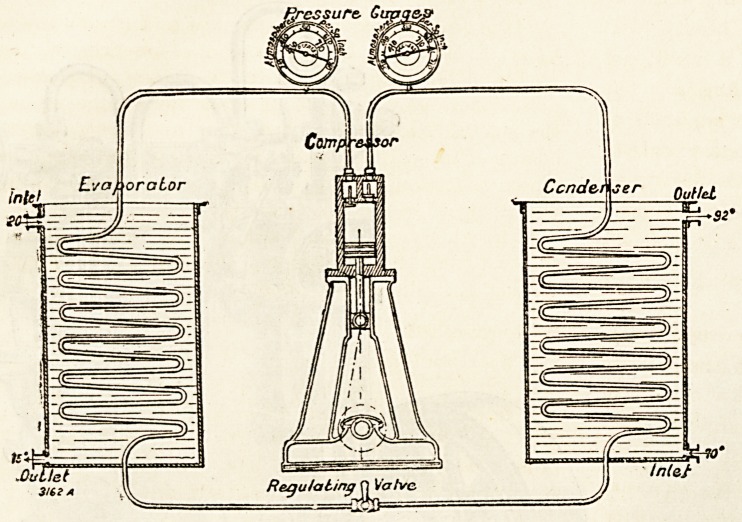


**Figure f2:**
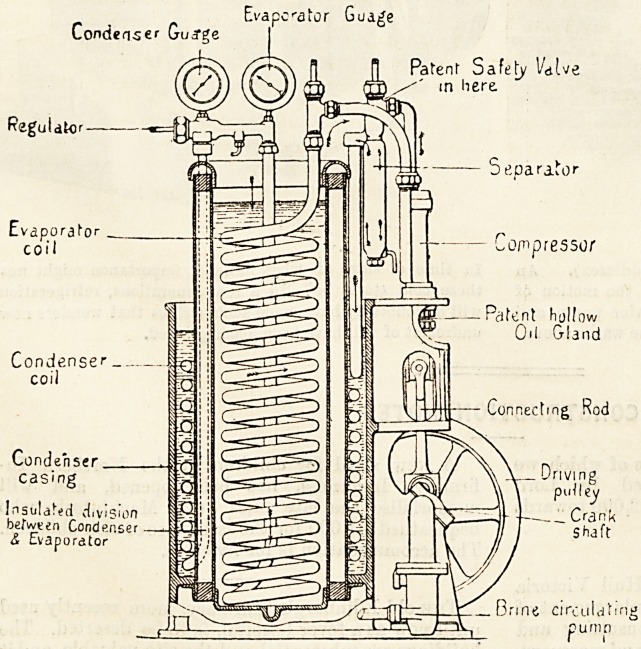


**Figure f3:**
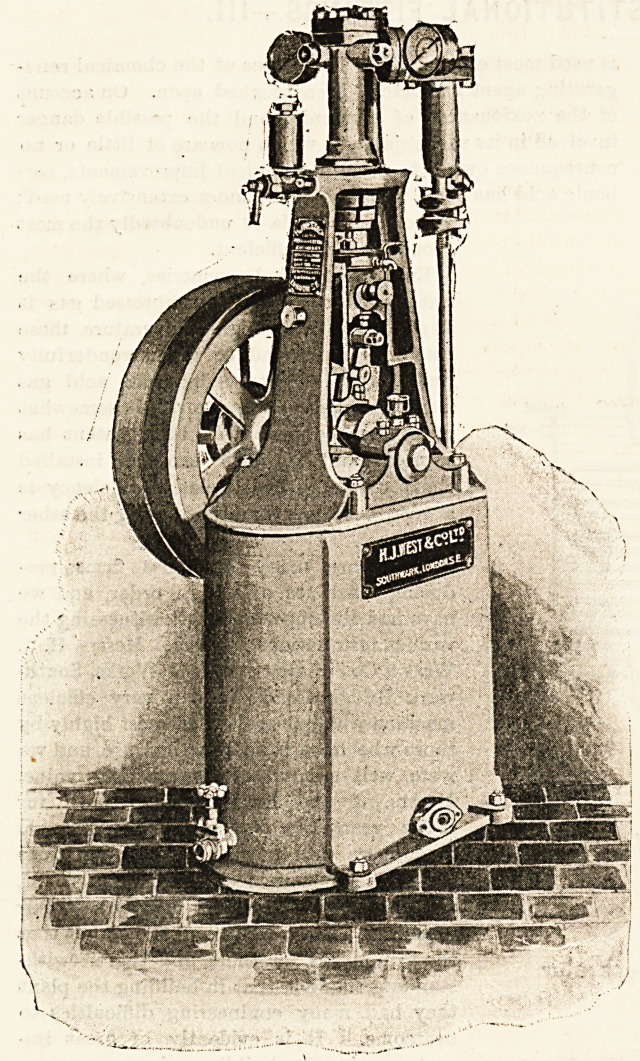


**Figure f4:**